# Self-phoretic Brownian dynamics simulations

**DOI:** 10.1140/epje/s10189-022-00177-3

**Published:** 2022-03-18

**Authors:** Sergi Roca-Bonet, Marisol Ripoll

**Affiliations:** Theoretical Physics of Living Matter, Institute of Biological Information Processing, Forschungszentrum Jülich, 52425 Jülich, Germany

## Abstract

**Abstract:**

A realistic and effective model to simulate phoretic Brownian dynamics swimmers based on the general form of the thermophoretic force is here presented. The collective behavior of self-phoretic dimers is investigated with this model and compared with two simpler versions, allowing the understanding of the subtle interplay of steric interactions, propulsion, and phoretic effects. The phoretic Brownian dynamics method has control parameters which can be tuned to closely map the properties of experiments or simulations with explicit solvent, in particular those performed with multiparticle collision dynamics. The combination of the phoretic Brownian method and multiparticle collision dynamics is a powerful tool to precisely identify the importance of hydrodynamic interactions in systems of self-phoretic swimmers.

**Graphic Abstract:**

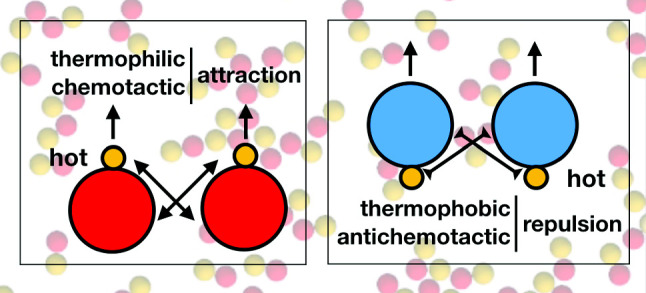

## Introduction

Computer simulation of active matter systems is currently a topic of intense scientific debate [1–5]. Active matter considers systems with at least one component able to draw energy from their environment in order to self-propel. Activity is an inherent property of most biological systems and recently a topic of growing interest for the investigation of synthetic active systems, with practical applications in fields such as microfluidics or microsurgery [6, 7]. In this line, phoresis is one of the main physical principles employed for the design of synthetic active matter. Phoresis refers to the drift that Brownian particles experience in the presence of a solvent with an intrinsic gradient, which becomes self-propulsion when the gradient is locally generated at the Brownian particle surface. Artificial microswimmers with a locomotion based on phoretic effects behave therefore as passive colloids unless activated via thermal [8–12], electric [13–17], chemical [18, 19], or magnetic [20–22] gradients.

The collective behavior of chemically propelled Janus particles showed aggregation behavior [18, 23, 24], and light powered micro-robots were observed to form living crystals [25–27]. The appearance of clustering and comet-like swarming structures was predicted by Brownian thermophilic active colloids [28, 29]. The system dimensionality [30, 31] and the presence and shape of hydrodynamic interactions have shown to play a relevant role on the collective behavior of such thermophilic swimmers [32, 33].

Janus-like phoretic particles have already been investigated by various simulation approaches, although not really compared with each other. Some of the approaches are purely Brownian, and self-phoretic propulsion is accounted simply by a constant impulse [28, 34, 35], or even a constant acceleration in systems that are supposed to increase their temperature on time [29]. In the absence of an explicit solvent, phoretic interactions between particles have been considered with an additional term, which might, or not, be coupled to the self-propulsion term. Thermal fluctuations are most frequently considered, and in a few cases also hydrodynamic interactions which are non-specific and typically only a far field approximation [36]. However, none of these methods completely accounts that self-phoretic Brownian swimmers propel with a well-defined Péclet number when isolated, while in the neighborhood of others, their velocity and interparticle interactions need to adjust to the actual distribution of heat sources. Different types of approaches consider the presence of an explicit solvent, such that phoretic effects arise in the presence of temperature or concentration gradients. This is the case of simulations performed with molecular dynamics [37], or dissipative particle dynamics [38], or with the mesoscopic simulation approach known as multiparticle collision dynamics (MPC) [11, 39]. With these approaches, the details of self-propulsion, inter-colloidal phoretic interactions, and hydrodynamic interactions are not directly imposed or tuned, but a consequence of the solvent–colloid interaction, colloid shape, and solvent intrinsic inhomogeneities. Therefore, in the studies of collective properties of phoretic active systems, the effects of steric, phoretic, or hydrodynamic interactions occur all simultaneously, such that the contribution of each of them is most frequently not possible to be identified. In this way, the design of strategies to make them distinguishable is timely and highly desirable.

Here, we propose a modification of the standard Brownian simulation method for Janus dimers, in which the effect of a single phoretic force results into the self-propulsion of the dimer and interparticle interactions are included but without any hydrodynamic interactions. We refer to this method as phoretic Brownian dynamics (Ph-BD). Furthermore, the precise values of self-propulsion velocity, intensity of the interactions, and Péclet number can eventually be closely mapped to those of the MPC simulations to allow for a fair comparison of the results obtained with both methods. On the other hand, we discuss another two simpler Brownian dynamics types of approaches for dimers. One with only self-propulsion, and another with a constant self-propulsion and phoretic interaction. An example study of dense systems of self-thermophilic dimers is here performed. The comparison of these three Brownian methods also provides interesting conclusions about the interplay of phoretic attraction/repulsion, alignment, and motility-induced instabilities.

**Fig. 1:**
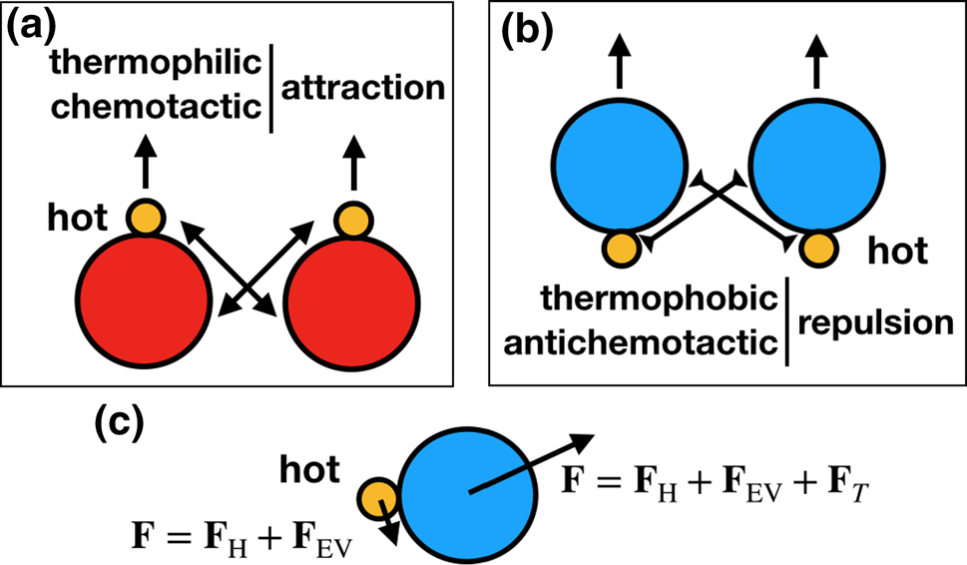
Sketches of the propulsion direction of self-phoretic asymmetric dimers and phoretic interaction between dimer pairs, which is: **a** attractive for the case of colloids drifting up gradient, and **b** repulsive in the opposite case. **c** Sketch of the implemented forces in the Ph-BD model

## Phoretic Brownian dynamics (Ph-BD)

Janus particles are characterized by having two different surface compositions. This is also the case of dumbbell-like structures in which each bead is made of a different material. For the sake of simplicity, we focus here mostly in the case of thermophoretic colloids, but the procedure is almost equivalent for other phoretic cases, in particular catalytic or diffusiophoretic ones. Thermophoretic dimers are made by one bead which is assumed to be at a higher temperature than the environment, mimicking a material with high heat conductivity which can be locally heated. The second bead is therefore exposed to a significant temperature gradient and responds to it depending on its intrinsic surface properties. In this way, dimers with a thermophilic (or chemotatic) behavior propel toward the hot bead (see Fig. 1a), while dimers with a thermophobic (or antichemotatic) behavior propel against the hot bead (see Fig. 1b). The same effect also controls the interaction between swimmers. Two thermophilic dimers swimming close to each other fell the temperature gradient produced not only by their own hot bead, but also by the hot bead of the neighboring dimer. This means that thermophilic dimers are attracted to neighboring dimers, while thermophobic dimers are mutually repelled from neighboring dimers, as depicted in Fig. 1a,b, which also exerts certain torques on the dimers. Therefore, in order to model phoretic active systems in a realistic manner, the effect of propulsion and interparticle interactions has to be included in a unified manner. Phoretic effects are related to the temperature gradients which vary locally.

Since the aim here is to describe the motion of colloids at low Reynolds numbers, we start by considering the overdamped Langevin equation [40, 41]

1 $$\begin{aligned} \dot{\mathbf {r}}_i(t) = \frac{\mathbf {F}_i(\mathbf {r})}{\mu _i} + \sqrt{\frac{2k_BT}{\mu _i}}\ \xi _i(t) \; , \end{aligned}$$

where $\mathbf {F}_i(\mathbf {r})$ is the total sum of forces acting on each particle *i*, and $\xi _i(t)$ is a random force with zero-mean $\langle \xi ^i_l(t) \rangle = 0$, and delta-correlated Gaussian $\langle \xi _{ik}(t)\cdot \xi ^{jl}(t^{\prime })\rangle = 2\gamma k_BT\; \delta (t-t^{\prime })\;\delta _{ij}\; \delta _{kl}$, with $k,l = x,y,z$ and $i,j=1,\ldots ,2N_s$ the particles under simulation, with $N_s$ the number of simulated dimeric swimmers. The friction coefficient, $\mu _i$, is considered to fulfill the Stokes–Einstein relation, $\mu _i= C_f\pi \eta s_i$, with $s_i$ the radius of the particle *i*, and $\eta$ the fluid viscosity. The numerical factor $C_f$ varies depending on colloid boundary conditions, and it is typically $C_f=6$ for stick and $C_f=4$ for slip boundary conditions [42]. The algorithm here used to integrate the motion equations was stochastic Euler. In general, the Euler algorithm has to be carefully considered due to its low precision and problems in most isothermal simulations [43, 44]. Nonetheless, the precision of this algorithm is sufficient for the study here performed, and other algorithms can be easily employed for more extensive investigations.

The details of the interactions are then provided by the forces, which distinguish two types of particles:

2 $$\begin{aligned} \mathrm{Phoretic:}&\mathbf {F}_{i} (\mathbf {r}_i) =\mathbf {F}_\mathrm{H,i} (\mathbf {r}_i) + \mathbf {F}_\mathrm{EV,i} (\mathbf {r}_i) + \mathbf {F}_\mathrm{T,i} (\mathbf {r}_i),\nonumber \\ \end{aligned}$$

3 $$\begin{aligned} \mathrm{Hot:}&\mathbf {F}_{j} (\mathbf {r}_j) =\mathbf {F}_\mathrm{H,j} (\mathbf {r}_j) + \mathbf {F}_\mathrm{EV,j} (\mathbf {r}_j), \end{aligned}$$

with $i,j = 1,\ldots ,N_s$ for the two beads of each dimer. The non-heated or phoretic bead is the one where the temperature gradient has a drift effect, which in this Ph-BD approach is considered in an effective manner by including a thermophoretic force $\mathbf {F}_T$ (see Fig. 1c). Meanwhile, the hot bead is considered to be at a higher but constant temperature, such that does not feel any thermophoretic force.

Pairwise interactions are considered, first the two beads forming each dimer are linked by a strong harmonic force $\mathbf {F}_\mathrm{H}$, obtained from the potential

4 $$\begin{aligned} U_\mathrm{H}(\mathbf {r}_{ij}) = \frac{\kappa _H}{2}\left( \mathbf {r}_{ij}-b\right) ^{2}, \end{aligned}$$

with the interparticle distance $\mathbf {r}_{ij} = \mathbf {r}_{i} - \mathbf {r}_{j}$, the harmonic constant $\kappa _H=10^4$, used to strongly fix the beads equilibrium distance *b* as the sum of the beads’ radii, $b=s_p + s_h$, with $s_p$ the radius of the phoretic bead and $s_h$ the radius of the hot bead. The relative dimensions of the dimer beads are defined by the radii aspect ratio, $\gamma =s_p/s_h$. Steric effects are accounted by the force $\mathbf {F}_\mathrm{EV,j}$, by which all non-linked beads interact with excluded volume interactions taken into account with the potential

5 $$\begin{aligned} U_\mathrm{EV}(\mathbf {r}_{ij}) = 4\varepsilon \left[ \left( \frac{\sigma }{\mathbf {r}_{ij}}\right) ^{2n} -\left( \frac{\sigma }{\mathbf {r}_{ij}}\right) ^{n} \right] +\epsilon , \end{aligned}$$

where *n* determines the potential softness, $\varepsilon =k_B\overline{T}$ relates to standard energy units, with $k_B$ the Boltzmann constant and $\overline{T}$ the average temperature, here both fixed to unity, defining the system units. The extra term on the right-hand side of the equation determines the repulsive character of the potential together with the cutoff radius, $r_c = 2^{1/n}\sigma$, and we use here $n=24$. The distance $\sigma$ simply relates to the sum of the radius of the two interacting beads.

The thermophoretic force exerted on the phoretic bead can be calculated as

6 $$\begin{aligned} \mathbf {F}_{T,i} (\mathbf {r}_i) = -\alpha _T k_B \mathbf {\nabla }T(\mathbf {r}_i)\; , \end{aligned}$$

where $\alpha _T$ is the bead thermodiffusion coefficient and $\nabla _{\mathbf {r}_i} T$ the gradient of temperature at the bead location. Note that $\alpha _T$ is a material property, which can be arbitrarily modified or chosen to match a value determined by experiments or by simulations with explicit solvent, as will be shown later.

The corresponding Laplace equations need to be solved to obtain a good estimation of the temperature gradient. We consider here three important and reasonable simplifications, such that the Laplace equation can be analytically solved: i) Each hot bead center acts as point-like heat source with temperature $T_h$ at the bead’s surface; ii) at a distance far enough the fluid reaches the average fluid temperature $\overline{T}$, taken as the reference unit, *i.e.,* $T(r\rightarrow \infty ) = \overline{T}$; iii) the effect of neighboring sources is considered to be additive. For each point-like source, all angular terms vanish due to the symmetry of the system such that $\nabla ^2 T(\mathbf {r}) = 0$, and the temperature at the center of each phoretic particle is then given by

7 $$\begin{aligned} T(r_i) = \sum _j \left( \frac{T_h - \overline{T}}{r_{ij}}\right) s_h + \overline{T}\; , \end{aligned}$$

where $r_{ij}=|r_i-r_j|$ and $r_j$ are the hot bead’s center positions. The expression in Eq. (7) corresponds to the gradient at the bead center. A more accurate estimation is to consider an effective value of the temperature gradient that considers the variation over the bead surface, for which Eq. (7) is integrated along the phoretic bead’s diameter. For a phoretic bead of radius $s_p$, placed at $r_i$, and with a hot bead placed at distance $r_{j}$, the integral limits are $r_{ij} - s_p$ and $r_{ij} + s_p$, such that the temperature gradient can be approximated by

8 $$\begin{aligned} \langle \nabla T\rangle (r_i) = \sum _j \frac{\left( \overline{T}-T_h\right) }{(r_{ij} + s_p)(r_{ij} - s_p)} s_h\; . \end{aligned}$$

Note that for an isolated swimmer the gradient is determined just by the linked hot bead, such that $r_{ij}=s_p+s_h$ is the only contributing term. For denser systems, the gradient takes into account the all neighboring hot heads, such that in the center of highly compact configurations the gradients eventually vanish and therefore also the thermophoretic force.

The dimer velocity $v_s$ and the rotational diffusion $D_r$ are therefore not direct inputs of the model, but indirectly determined from other input values, mainly $\alpha _T$, $\nabla T$, $s_p$, and $\gamma$. The value of the module of $v_s$ is given by

9 $$\begin{aligned} v_s = \frac{\alpha _T k_B \langle \nabla T\rangle }{\mu }. \end{aligned}$$

where both the dimer friction $\mu =C_f \pi \eta (s_h+s_p)$ and the temperature gradient $\langle \nabla T\rangle = (T_h-\overline{T})/(s_h+2s_p)$ depend on the hot and phoretic bead sizes. The axis direction of the swimmer $\mathbf {n}$ aligns with the direction of the temperature gradient direction considering also the sign of $\alpha _T$, which determines the direction of $v_s$. The self-propulsion velocity can also be obtained from the simulations as $v_s = \mathbf {v}\cdot \mathbf {n}$. The rotational diffusion $D_r$ depends mostly on the particle size and aspect ratio $\gamma$ and can be obtained by characterizing the longtime behavior of the mean-squared angular displacement, $\varDelta \mathbf {e}^2 =\langle (\mathbf {e}(t)-\mathbf {e}(t^{\prime }))^2\rangle$, in simulations with equilibrium conditions; this is with $T_h=\overline{T}$. The resulting Péclet number can then be defined as $\text {Pe} = v_s/(D_rs_p)$.

## Other active Brownian dimer models

The method proposed in this manuscript, Ph-BD, differs from other approaches employed in the literature in the way that phoretic self-propulsion and interparticle phoretic interactions are coupled to each other. In order to better understand the relevance of this coupling, we propose two alternative methods. Self-propelled spherical colloids have been extensively investigated with the so-called active Brownian particle (ABP) model [34, 45], which simply assumes a constant propulsion velocity in the particle main axis. The physical origin of the propulsion is not specified, such that it could be phoretic but also any type of biological specificity. We adapt this idea to the dimeric case by considering $N_s$ swimmers with two bounded monomers each, where the hot bead just follows Eq. (3), and the phoretic bead

10 $$\begin{aligned} \mathbf {F}_{i} (\mathbf {r}_i) =\mathbf {F}_\mathrm{H,i} (\mathbf {r}_i) + \mathbf {F}_\mathrm{EV,i} (\mathbf {r}_i) + \mu v_s\mathbf {n}, \end{aligned}$$

where the friction is that of the dimeric structure $\mu =C_f \pi \eta (s_h+s_p)$ and $\mathbf {n}$ is the orientation vector of the dimer. We here call this method the *active Brownian multimer model* (ABM). With this approach, there is no additional interparticle interactions, such that all apparent repulsions or attractions are consequence of the propulsion and/or steric interactions.

The second approach includes also the effect of the phoretic interaction with a force as given in Eq. (6), but considering only the heat sources of neighboring hot beads,

11 $$\begin{aligned} \mathbf {F}_{i} (\mathbf {r}_i) =\mathbf {F}_\mathrm{H,i} (\mathbf {r}_i) + \mathbf {F}_\mathrm{EV,i} (\mathbf {r}_i) + \mu v_s \mathbf {n} -\alpha _T k_B \mathbf {\nabla }T(\mathbf {r}_i)\;\nonumber \\ \end{aligned}$$

where the temperature gradient can be calculated with Eq. (7) or Eq. (8). We refer to this method the *active Brownian multimers with phoresis model* (ABM+ph). With this approach, the phoretic interdimer attraction (or repulsion) is in principle decoupled from the dimer propulsion since there are two different parameters control, *i.e.,* $v_s$ and $\alpha _T$. There are approaches in which these, or very strongly related parameters, are independently varied [34, 35], which cannot really correspond to a phoretic model since both self-propulsion and interparticle phoresis are simultaneously originated. Besides the fact that $v_s$ and $\alpha _T$ should be related by Eq. (9) for thermophoresis, or an equivalent one for other phoretic phenomena, there is another relevant difference between Ph-BD and ABM+ph which is that in ABM+ph, the velocity of the particles is fixed, namely it does not depend on the position of the neighboring particles, while for Ph-BD both the velocity of the particle and the interparticle interactions are damped when various other swimmers are in the neighborhood, as accounted in the temperature gradient calculation. In order to better understand this effect, we focus here in the case that $v_s$ and $\alpha _T$ are linked by Eq. (9).

## Hydrodynamic self-phoretic model

The methods introduced until now consider steric, stochastic, and phoretic interactions, which means that hydrodynamic interactions (HI) have been disregarded. Although in some cases this can be clearly justified, the effect of HI is frequently not known. In order to provide a tool that allows for a fair comparison, we consider now the method known as multiparticle collision dynamics (MPC) [11, 39]. Multiparticle collision dynamics is here used to simulate the explicit solvent particles and their interactions [46, 47], while molecular dynamics (MD) is employed for colloid–colloid and colloid–solvent interactions. This hybrid MPC-MD approach has already extensively proved to include both hydrodynamics and phoretic effects [48–51].

***MPC method for the solvent*** The MPC method considers the solvent composed of *N* point particles of mass *m* performing alternate streaming and collision steps. During the streaming step, fluid particles translate ballistically for a certain time, *h*, the *collision time*, this is $\mathbf {r}_k(t+h) = \mathbf {r}_k(t) + h\mathbf {v}_k(t)$. In the collision step, the particles are binned into cubic cells of side *a*, with a grid shift applied to the binning in order to restore Galilean invariance [52]. Interparticle interactions are treated within each of these cells, in which particles interchange linear momentum with all other particles in the same cell. Here, we employ the stochastic rotational dynamics collision rule, in which the momentum interchange is made rotating by an angle $\alpha$ the relative velocities to the center of mass around a random axis on the cell, $\mathbf {v}_i(t+h) = \mathbf {v}_{cm,i}(t) + \mathbf{R}(\alpha )\left[ \mathbf {v}_i(t) - \mathbf {v}_{cm,i}(t) \right]$, with $\mathbf{R}(\alpha )$ the rotation matrix, $i=1,\ldots ,N$ the particle index, and $\mathbf {v}_{cm,i}$ the center of mass velocity of the cell where particle *i* was sorted, such that linear momentum and kinetic energy are conserved in each collision cell. Simulation units are defined by the choice of $a=1=m=k_B\overline{T}$, with which we rescale all quantities in this work. In the simulations here performed, the fluid properties are determined by the values of the collision angle $\alpha =120^{\circ }$, the average fluid particles per cell $\rho =10$, and the collision time, $h=0.1$. With these choices, the solvent diffusion coefficient is $D = 0.06$, the kinematic viscosity $\nu = 0.79$, and the thermal diffusivity $\kappa _T= 0.15$ [53–56]. The comparison with specific solvents can be done via dimensionless numbers, mainly the Schmidt number, $\text {Sc}=\nu /D = 13$, and the Prandtl number, $\text {Pr}=\nu /\kappa _T=5.3$. While $\text {Sc}$ is smaller than the value for water, $\text {Pr}$ is quite close to it. These two values ensure that momentum transfer is faster than that of mass, providing an efficient way to include hydrodynamic interactions, and that the stability of local temperature gradients is also ensured.

***Molecular dynamics*** Fluid–colloid interactions are considered using molecular dynamics, with the equations of motion being integrated using the velocity Verlet algorithm [43, 44, 57]. The thermophoretic nature of the colloids is determined by the choice of the fluid–colloid interactions, for which we used a displaced Mie-like potential,

12 $$\begin{aligned} U_\mathrm{FC}(r) = 4\varepsilon \left[ \left( \frac{\sigma }{r-\varDelta }\right) ^{2n} - \left( \frac{\sigma }{r-\varDelta }\right) ^{n} \right] +C.\qquad \end{aligned}$$

This potential is very similar to that in Eq. (5) with the introduction of the $\varDelta$ and the *C* parameters. The bead size is now determined by $s \equiv \sigma + \varDelta$, where $\varDelta$ can be understood as the size of a core with hard-sphere interactions and $\sigma$ the size of an additional layer with repulsive potential interactions. In this work, we use $\sigma = \varDelta$ and $s_p=6$ for the size of the phoretic bead. The extra term on the right-hand side of Eq. (12) is $C=\epsilon$ for repulsive interactions, which have proved to account for thermophilic colloidal behavior, and $C=0$ for attractive interactions for thermophobic [58, 59]. For these interactions, $n=3$ is chosen to obtain a soft repulsive potential for the phoretic (philic) bead, whereas $n=24$ is chosen for the heated particle and also for the attractive potential (phobic). The cutoff radius of the interactions is $r_c = 1.26\sigma +\varDelta$ for the repulsive potential and $r_c=1.1\sigma +\varDelta$ for the attractive. Harmonic and excluded volume interactions are considered similarly as for the Ph-BD case with Eq. (4) and Eq. (5). In order to mimic the heating produced by laser illumination of partially gold-coated colloids [60], we have rescaled the temperature of the fluid within a small shell (of $0.08s_h$) around the heated bead to $T_h > \overline{T}$, while cooling the average temperature of the whole system to $\overline{T}=1$, by means of a simple velocity rescale [55, 60]. Unless otherwise specified, we use $T_h = 1.5$. All colloid–colloid interactions have been implemented via Eq. (5); this is Eq. (12), with $\varDelta =0$, $\sigma = s$ and $n=24$, with the interactions being cut at $r_c = 2^{1/24}\sigma$.

This method has been implemented on LAMMPS [61], where we have modified the “srd” package routine [62] to include the colloid–solvent potential interactions. The MD time step has been chosen as $\varDelta t=0.01h$, similar as in the Brownian simulations, and the mass *M* of the colloidal beads is chosen to make the colloids neutrally buoyant.

***Parameters for the comparison MPC*** *vs.* ***Ph-BD*** In order to perform a fair comparison of the methods with and without HI, we are interested in having systems as similar as possible. Some values are input parameters in the Brownian dynamics simulations and therefore very easy to match, such as the average temperature $k_B\overline{T}=1$, or the fluid viscosity $\eta =\nu \rho =7.9$. The numerical factor $C_f$ for the friction coefficient is fixed as $C_f = 3$ in order to match the employed MPC-SRD algorithm without angular momentum conservation and slip boundary conditions [63, 64]. Other parameters are not direct input and need to be more carefully considered. For a proper comparison, it is of importance that parameters chosen for the two simulations models result in matching self-propulsion velocity and the Péclet number of diluted swimmer dimers systems. For this, we need to characterize the simulated thermophoretic coefficient $\alpha _T$ and rotational diffusion $D_r$.

**Fig. 2:**
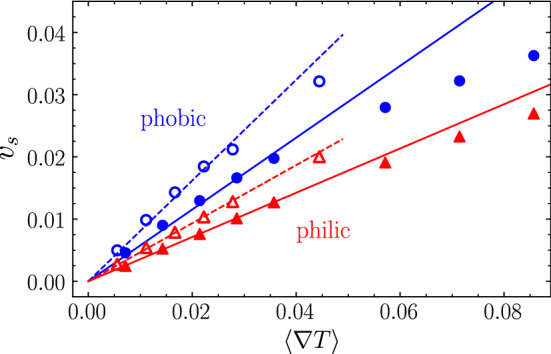
Self-propulsion velocity $v_s$ of single dimers simulated with MPC as a function of the temperature gradient $\langle \nabla T\rangle$ felt by the phoretic bead, for various dimer types. Results for dimers with phoretic bead $s_p=6$. Circles (in blue) correspond to thermophobic dimers; triangles (in red) correspond to thermophilic dimers. Full symbols correspond to asymmetric dimers ($\gamma =3$); empty symbols to symmetric dimers ($\gamma =1$). Lines relate to linear fits to Eq. (9) for small gradients

The thermophoretic coefficient $\alpha _T$ of a spherical bead could in principle be determined in full hydrodynamic simulations with an external temperature gradient [58]. This would be, however, a too rough estimation, first because the constant and position-dependent gradients are different, and second because it is known that the proximity of the hot bead screens part of the phoretic interactions of the surrounding solvent and the colloid surface. A more adequate estimation can be done by measuring the self-propelled velocity of a single swimmer with Eq. (9) and relating it then to the thermophoretic coefficient $\alpha _T$. Figure 2 shows simulation results for four types of dimeric swimmers, corresponding to thermophobic and thermophilic character, and to the symmetric ($\gamma =1$) and asymmetric ($\gamma =3$) geometries. Velocities are calculated as an average of 20 independent simulations, and the error bars are of the order of the symbol size. Simulations are performed at various temperature gradients, which are achieved by changing the temperature of the hot bead $T_h$. The increase in the velocity is clearly linear for moderate gradients, which allows us to determine the value of the thermophoretic coefficient for all the investigated cases as shown in Table 1. For the largest temperature gradients, the velocities deviate from the linear behavior. This deviation from the Fourier linear behavior can be expected and here can also be related to the limit of the method for these temperature gradients. Note that the negative sign of $\alpha _T$ is well established by convention and it refers the motion of the swimmer toward the heat source. The sign of this coefficient naturally induces the interdimer phoretic attraction for thermophilic dimers, and phoretic repulsion for thermophobic ones, as shown in Fig. 1a, b, such that no further assumption has to be made in this regard.

**Table 1:** Thermophoretic coefficient $\alpha _T$ of single thermophilic and thermophobic self-propelled dimers, with different aspect ratios $\gamma =s_h/s_p$, as obtained from MPC simulations. Values are obtained as a fit to the data in Fig. 2 to the expression in Eq. (9) for small temperature gradients. Values of the self-propelled velocity $v_s$ and rotation diffusion coefficient $D_r$ and Péclet number Pe calculated as explained in the text

	Phobic		Philic	
	$\gamma =3$	$\gamma =1$	$\gamma =3$	$\gamma =1$
$\alpha _T$	345	725	−213	−418
$v_s\ (\times 10^2)$	1.95	2.20	1.27	1.28
$D_r\ (\times 10^5)$	9.06	2.04	9.2	1.9
Pe	36	180	23	112

**Fig. 3:**
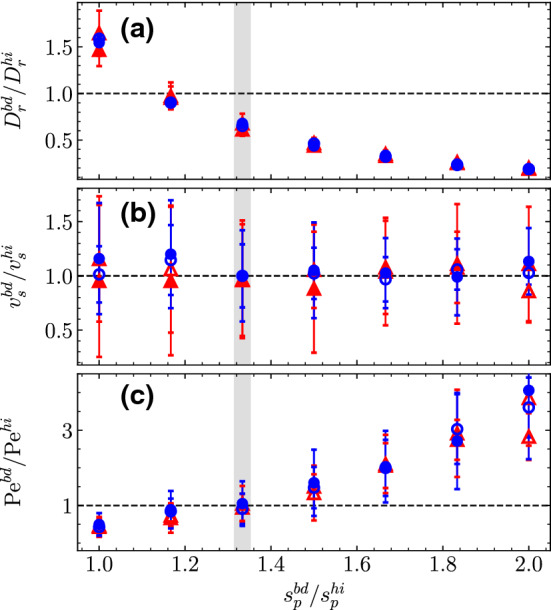
Dynamical quantities measured with single-dimer Ph-BD simulations as a function of the phoretic bead size and normalized by the values of the hydrodynamic simulations. **a** Rotational diffusion coefficient $D_r$, **b** self-propulsion velocity $v_s$, **c** resulting Péclet number Pe. All quantities are normalized by the reference values obtained for hydrodynamic simulations with $s_p^{hi}=6$, in Table 1. The dashed lines at unity indicate perfect agreement between Ph-BD and MPC simulations. Thick vertical gray line corresponds to the case with optimal agreement for both $v_s$ and Pe, which occurs for $s_p^{bd}=8$

To perform simulations with Ph-BD and MPC of dimers with the same bead sizes, the same solvent input parameters, and the same $\alpha _T$ seems then a good strategy to have a fair comparison between methods, only the Péclet number is left to be discussed. The values of $D_r$ for the specified values result to be close to $60\%$ larger in the Ph-BD simulations than in those performed with MPC, for all the four investigated cases (see Fig. 3a for the case $s_p^{bd}/s_p^{hi}=1$). This is because the rotational diffusion is not a parameter fixed in any of the two methods but a consequence of all the other parameters, such as friction, particle size, thermophoretic coefficient, or fluid particle interactions which are different in both methods. In order to modify the rotational diffusion coefficient without affecting other characteristic values, it is possible to vary the overall bead sizes. Further simulations in equilibrium with dimers with different $s_p$ (and different $s_h$ to preserve $\gamma$) are performed, and the measured values of $D_r$ are shown in Fig. 3a. As expected, the results show a decay of $D_r$ for growing particle sizes, which is produced just by the thermal noise in the position update of the two linked dimer monomers. Using a different monomer size allows then the tuning of the rotational diffusion, but, for simulations out of equilibrium with a fixed value of $T_h$, also modifies the temperature gradient at the phoretic monomer surface and therefore also the resulting self-propelled velocity. The solution to keep the same $v_s$ value is then to modify $T_h$ to keep the gradient constant when changing the monomer size. As a check of this principle, we perform simulations modifying both $s_p$ and $T_h$ for a given gradient and then measure the self-propelled velocity. The results are shown in Fig. 3b in comparison with those of the self-propelled velocity of the hydrodynamic simulations used here as an input, and the agreement is very good within the error of the measurements in all cases. Note that in order to keep the same $v_s$ value, to modify $T_h$ is equivalent to modify $\alpha _T$ since it is the product of both which determines $F_T$ and $v_s$, as shown in Eq. (6) and Eq. (9), respectively. The resulting Péclet number shown in Fig. 3c results in a growing trend with particle size, which is related to the variation in the rotational diffusion. From Fig. 3, it is also clear that the optimal value is given by Brownian simulations with $s_p=8$, such that all presented Brownian simulations are from now on carried with this value.

## Comparative study for collective dynamics

In order to perform a comparative study of the Brownian methods, simulations of dimeric thermophilic swimmers are performed first with the three Brownian methods previously discussed. Ensembles of 200 dimers both asymmetric, $\gamma =3$, and symmetric, $\gamma =1$, have been studied for a quasi-2d confinement case. In principle, this refers to 3d slides of liquid in which the swimmers move on a plane, which for the Brownian dynamics simulations means that the motion occurs in two dimensions.

**Fig. 4:**
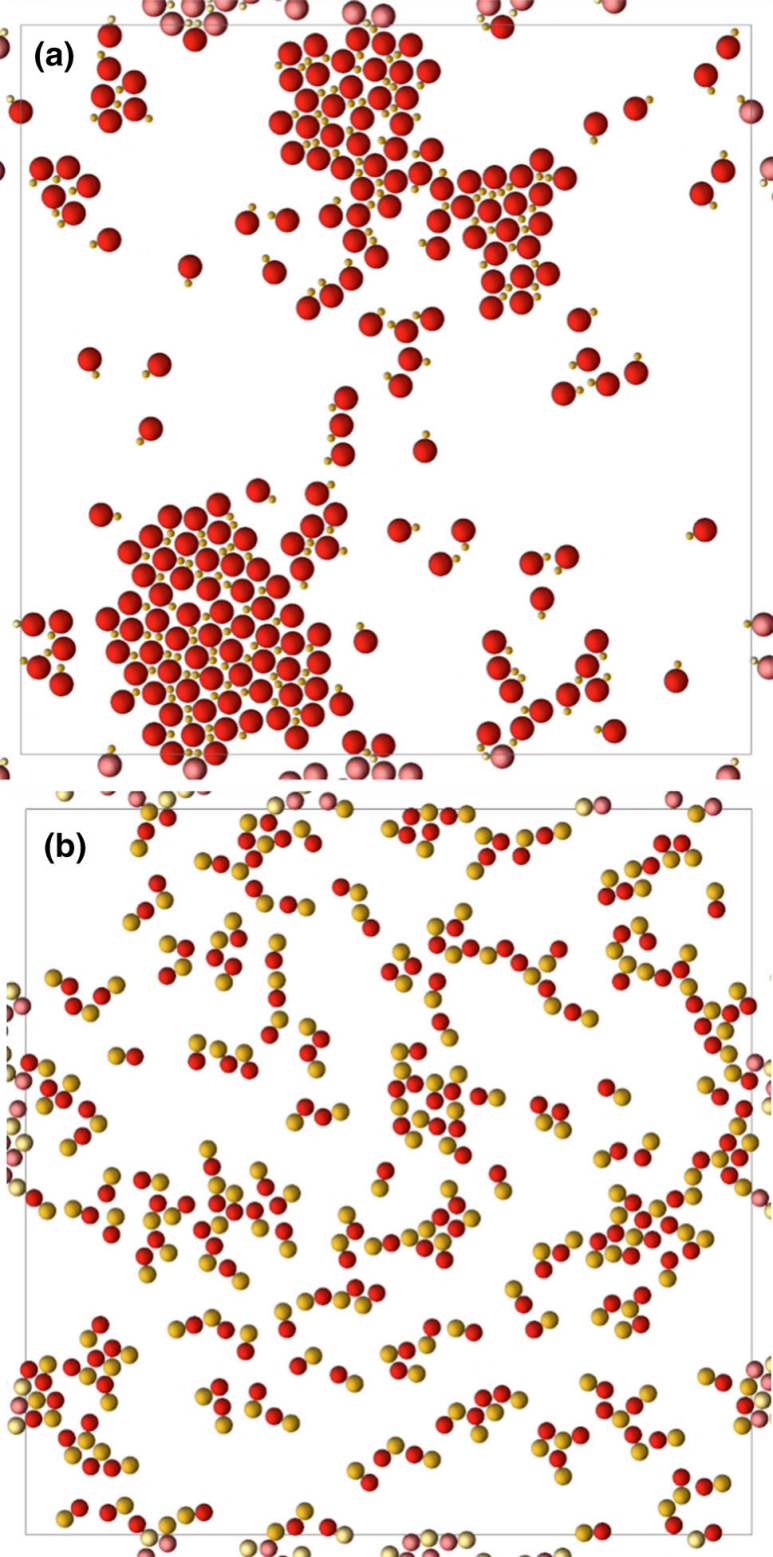
Snapshots of ensembles of 200 self-thermophilic swimmers at times around $300\tau _b$ for density $\phi =0.2$ simulated with the Ph-BD method. **a** Asymmetric dimers ($\gamma =3$), showing a few large stable clusters; **b** symmetric dimers ($\gamma =1$), showing a number of small transient clusters

The configuration used to initialize the simulation has the dimers center of mass placed on a square lattice covering almost the whole simulation box, with a randomly chosen direction of the dimer axis. Initial order disappears very quickly in all cases. All simulations run for a time $t\sim 300\tau _b$, with $\tau _b$ the ballistic time of a swimmer, defined as $\tau _b = s_p/v_s$, and representative snapshots of the latest configurations are shown in Fig. 4. Asymmetric dimers propel forming small clusters; some of these clusters dissolve due to collisions with isolated dimers or pairs of dimers, and some other coalesce with other small cluster, forming larger and more stable clusters, as can be seen in Fig. 4a. Symmetric dimers show initially similar dynamics, although interestingly the large clusters do not become stable and also end up dissolving in this case, as can be seen due to the small-sized clusters in Fig. 4b. Snapshots in Fig. 4 correspond to simulations performed with the Ph-BD method; qualitative roughly similar results are also obtained with ABM and ABM+ph methods. In order to more precisely understand the involved mechanisms and the difference between the methods, the quantification of a dynamic quantity is employed.

We introduce here the calculation of bounding time $\tau _c$ for both asymmetric and symmetric dimers at two density values. This bounding time is defined as the average time that encounters of dimers pairs remain at maximum colloid surface to colloid surface distance $0.75s_p$ of each other. This bounding time is obtained as a time average and as an average over five independent simulations. In this way, particles forming unstable clusters have a well-defined average bounding time that might be longer or shorter depending on the cluster instability. Particles inside a stable cluster have a theoretically infinite bounding time, which in our measurements shows as a quantity growing with the simulation time.

**Fig. 5:**
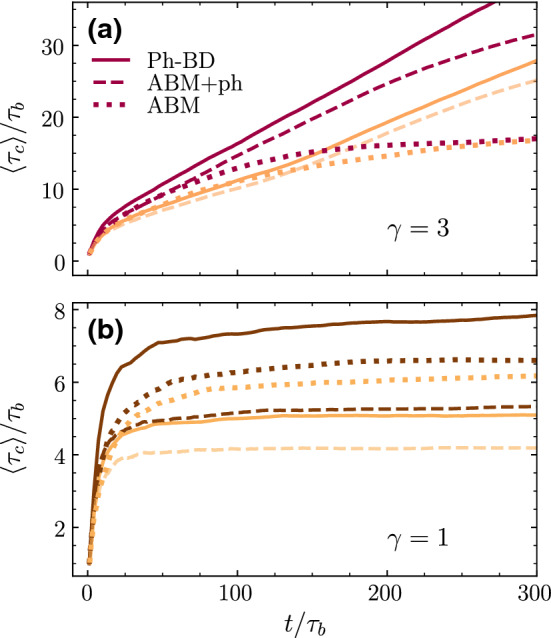
Bounding time $\tau _c$ calculated as a time average and shown here as a function of simulation time normalized with the dimer ballistic times $\tau _b$. Results for simulations with the three Brownian dynamics methods for 200 thermophilic dimers distinguished by the inset labels. Results at densities $\phi =0.2$ are shown with light colors, while results with $\phi =0.3$ are displayed with darker colors. **a** Asymmetric dimers and **b** symmetric dimers

The bounding times of asymmetric dimers in Fig. 5a show to form stable clusters at both densities in simulations with phoretic attraction; this is with Ph-BD and ABM+ph simulations. Simulations without phoretic interparticle attraction show to saturate to a constant value, which curiously is the same for both simulated densities. This means that for these asymmetric dimers at these densities, self-propulsion is not enough to stabilize the clusters, and the consideration of the corresponding phoretic attraction stabilizes the clusters. The bounding times are slightly smaller for ABM+ph with respect to Ph-BD. Although the difference is not large, it indicates that diminishing the propulsion velocity of the dimers inside the cluster slightly increases its cohesion. The bounding times of symmetric dimers in Fig. 5b show that all three Brownian methods form just unstable clusters; several interesting conclusions can still be drawn from these results. The first one relates to the effect of density, which shows to increase the bounding time in all cases, although with different intensity. Density increases the probability of encounters which has two opposite effects since it enhances both clustering formation and its dissolution. For the symmetric dimers without phoresis (ABM), the effect is small but clear at not too small times. This is in contrast to the asymmetric case for which the difference is much smaller and even seem to disappear for large averaging times. We relate this difference to the particle-induced alignment when two dimers collide, effect that is much larger for symmetric dimers. Another clear conclusion is that Ph-BD enhances stability with respect to ABM+ph and that this effect increases with density. This is again related to the fact that the smaller propulsion velocity of the dimers inside a cluster for the Ph-BD cases increases their stability. The effect is though not straightforward to predict since it is not shown to be much larger for asymmetric dimer swimmers where the formed clusters are larger, than in the case of symmetric dimers where only small clusters would be affected. Curious is also the difference between ABM and ABM+ph for the symmetric dimers. The collective simulations here presented only analyze the thermophilic case, such the inclusion of phoresis includes an interparticle attraction, expected to translate into larger clustering affinity. This is indeed the case for the asymmetric dimers, but not for the case of symmetric dimers. Phoretic attraction combined with the non-adjusting self-propulsion velocity seems to induce additional alignment of the symmetric dimers, such that they became more prompted to swim away from the small nucleated clusters, producing this somehow counterintuitive effect. In other words, the fact that ABM dimers do not significantly change their orientation when colliding with others makes that in some cases they get stuck in configurations longer than in the presence of attraction, providing such structures with additional stability. For the $\phi =0.3$ symmetric density case, the ABM simulations have almost the same stability properties as those with Ph-BD, while for $\phi =0.2$, ABM simulations are even more stable than those with Ph-BD, where both self-propelled velocity and attraction diminish in the neighborhood of other dimers.

Simulations in the collective regime with the hydrodynamics phoretic model MPC are also performed in the previously discussed regimes. The most relevant conclusion is the occurrence of qualitative differences with the systems here presented, which, due to the fair comparison that these methods provide, can be related just to hydrodynamics. Detailed understanding of such results requires a detailed discussion of the shape of the hydrodynamic fields which will be presented elsewhere.

## Summary and discussion

The Ph-BD method to perform Brownian dynamics simulations with a realistic inclusion of the phoretic self-propulsion and interactions is here presented. The main idea is that simply considering the well-known dependence of the phoretic force with the applied gradient properly couples the self-propelled velocity and the interaction between two or more particles. The Ph-BD method is here compared with other two simpler versions of BD simulations, which draws interesting conclusions illustrating the very subtle interplay of self-propulsion, phoretic-induced attraction, repulsion, or orientation. Depending on the particle geometry, properties, and overall densities, these effects show that they can act together, or against each other. We also show in detail how the Ph-BD method can be adjusted to map the properties of experimental systems or simulations with explicit solvent, as illustrated here for MPC simulations. The combination of MPC and Ph-BD simulations offers therefore the possibility to compare simulated systems which only differ in the inclusion of solvent-mediated interactions, which is a very powerful tool to understand the effect of hydrodynamic interactions. These methods are here used to investigate the properties of thermophoretic dimers, but it can be almost trivially extended to other phoretic effects such as diffusiophoresis, and also to other multimeric structures such as trimers or other oligomeric swimmers. Preliminary analysis indicates that it can also be extended to Janus spherical particles. The presented results for the collective dynamics of thermophoretic swimmers also indicate that these are the basis of synthetic active materials with various perspectives for applied materials.

## References

[1] Gompper G, Winkler RG, Speck T, Solon A, Nardini C, Peruani F, Löwen H, Golestanian R, Kaupp UB, Alvarez L (2020). J. Phys.: Condens. Matter.

[2] C. Bechinger, R. Di Leonardo, H. Löwen, C. Reichhardt, G. Volpe, Rev. Mod. Phys. **88** (2016)

[3] G. De Magistris, D. Marenduzzo, Physica A **418** (2015)

[4] M.C. Marchetti, J.F. Joanny, S. Ramaswamy, T.B. Liverpool, J. Prost, M. Rao, A.S. Simha, Rev. Mod. Phys. **85** (2013)

[5] S. Ramaswamy, Annu. Rev. Condens. Matter Phys. **1** (2010)

[6] Paxton WF, Kistler KC, Olmeda CC, Sen A, StAngelo SK, Cao Y, Mallouk TE, Lammert PE, Crespi VH (2004). J. Am. Chem. Soc..

[7] Elgeti J, Winkler RG, Gompper G (2015). Rep. Prog. Phys..

[8] Piazza R, Parola A (2008). J. Phys. Condens. Matter.

[9] Jiang HR, Yoshinaga N, Sano M (2010). Phys. Rev. Lett..

[10] A. Würger, Rep. Prog. Phys. **73** (2010)

[11] Yang M, Ripoll M (2011). Phys. Rev. E.

[12] Burelbach J, Zupkauskas M, Lamboll R, Lan Y, Eiser E (2017). J. Chem. Phys..

[13] Morrison FA (1970). J. Colloid Interface Sci..

[14] Makino K, Ohshima H (2010). Langmuir.

[15] Hatlo MM, Panja D, van Roij R (2011). Phys. Rev. Lett..

[16] Sabass B, Seifert U (2012). J. Chem. Phys..

[17] Samin S, van Roij R (2017). J. Fluid. Mech..

[18] Kapral R (2013). J. Chem. Phys..

[19] Saha S, Golestanian R, Ramaswamy S (2014). Phys. Rev. E.

[20] Suwa M, Watarai H (2011). Anal. Chim. Acta.

[21] Benelmekki M, Martinez LM, Andreu JS, Camacho J, Faraudo J (2012). Soft Matter.

[22] Sherman ZM, Pallone JL, Erb RM, Swan JW (2019). Soft Matter.

[23] Theurkauff I, Cottin-Bizonne C, Palacci J, Ybert C, Bocquet L (2012). Phys. Rev. Lett..

[24] Liebchen B, Marenduzzo D, Pagonabarraga I, Cates ME (2015). Phys. Rev. Lett..

[25] Ibele M, Mallouk T, Sen A (2009). Angewandte Chemie International Edition.

[26] Palacci J, Sacanna S, Steinberg AP, Pine DJ, Chaikin PM (2013). Science.

[27] Mallory SA, Valeriani C, Cacciuto A (2018). Annual Rev. Phys. Chem..

[28] Golestanian R (2012). Phys. Rev. Lett..

[29] Cohen JA, Golestanian R (2014). Phys. Rev. Lett..

[30] Stenhammar J, Marenduzzo D, Allen RJ, Cates ME (2014). Soft Matter.

[31] Wysocki A, Winkler RG, Gompper G (2014). EPL.

[32] Delfau J-B, Molina J, Sano M (2016). EPL.

[33] Wagner M, Roca-Bonet S, Ripoll M (2021). Eur. Phys. J. E.

[34] Pohl O, Stark H (2014). Phys. Rev. Lett..

[35] Pohl O, Stark H (2015). Euro. Phys. J. E.

[36] Saha S, Ramaswamy S, Golestanian R (2019). New J. Phys..

[37] Kroy K, Chakraborty D, Cichos F (2016). Euro. Phys. J. Spec. Topics.

[38] D.A. Fedosov, A. Sengupta, G. Gompper, Soft Matter **11**, 6703 (2015) 10.1039/c5sm01364j 26223678

[39] Rückner G, Kapral R (2007). Phys. Rev. Lett..

[40] P. Langevin, C. R. Acad. Sci. Paris **146** (1908)

[41] R. Pathria, *Statistical Mechanics* (1972)

[42] Hansen JP, McDonald IR (2013). Theory of simple liquids.

[43] Frenkel D, Smit B (1996). Understanding molecular simulations.

[44] M.P. Allen, *Introduction to Molecular Dynamics Simulation* (John von Neumann Institute for Computing, Jülich, 2004), Vol. 23 of *NIC*

[45] Speck T (2016). Eur. Phys. J. Spec. Top..

[46] Malevanets A, Kapral R (1999). J. Chem. Phys..

[47] Malevanets A, Kapral R (2000). J. Chem. Phys..

[48] Kapral R (2008). Adv. Chem. Phys..

[49] G. Gompper, T. Ihle, D. Kroll, R. Winkler, *Advanced Computer Simulation Approaches for Soft Matter Sciences III* (Springer Berlin Heidelberg, 2009), Vol. Advances in Polymer Science, 221 of *Advanced Computer Simulation Approaches for Soft Matter Sciences III*, pp. 1–87

[50] Mussawisade K, Ripoll M, Winkler RG, Gompper G (2005). J. Comp. Phys..

[51] Yang M, Ripoll M (2013). Soft Matter.

[52] Ihle T, Kroll DM (2001). Phys. Rev. E.

[53] Tüzel E, Ihle SM, Kroll DM (2003). Phys. Rev. E.

[54] Tüzel E, Ihle T, Kroll DM (2006). Phys. Rev. E.

[55] Lüsebrink D, Ripoll M (2012). J. Chem. Phys..

[56] Ripoll M, Mussawisade K, Winkler RG, Gompper G (2005). Phys. Rev. E.

[57] Verlet L (1967). Phys. Rev..

[58] Lüsebrink D, Yang M, Ripoll M (2012). J. Phys.: Condens. Matter.

[59] Wagner M, Ripoll M (2017). EPL.

[60] H. Jiang, T. Kiørboe, J. Exp. Biol. **214**(3) (2011) 10.1242/jeb.049288 21228207

[61] S. Plimpton, J. Comput. Phys. **117** (1995)

[62] M.K. Petersen, J.B. Lechman, S.J. Plimpton, G.S. Grest, P. Jin’t Veld, P.R. Schunk, J. Chem. Phys. **132**, 174106 (2010) 10.1063/1.3419070 20459155

[63] Pooley CM, Yeomans JM (2005). J. Phys. Chem. B.

[64] Yang M, Theers M, Hu J, Gompper G, Winkler RG, Ripoll M (2015). Phys. Rev. E.

[65] J.S. Centre, Journal of large-scale research facilities **2** (2016)

